# Correction: Performance of novel antibodies for lipoarabinomannan to develop diagnostic tests for *Mycobacterium tuberculosis*

**DOI:** 10.1371/journal.pone.0297828

**Published:** 2024-01-23

**Authors:** Jason L. Cantera, Lorraine M. Lillis, Roger B. Peck, Emmanuel Moreau, James A. Schouten, Paul Davis, Ruixiang B. Zheng, Todd L. Lowary, Paul K. Drain, Alfred Andama, Abraham Pinter, Masanori Kawasaki, Gunilla Källenius, Christopher Sundling, Karen M. Dobos, Danara Flores, Delphi Chatterjee, Eileen Murphy, Olivia R. Halas, David S. Boyle

Ruixiang Blake Zheng and Todd L. Lowary are not included in the author byline.

Ruixiang Blake Zheng should be listed as the seventh author and affiliated with the Department of Chemistry, University of Alberta, Edmonton, Alberta, Canada T6G 2G2. The contributions of this author are as follows: Data Curation, Formal Analysis, Investigation and Methodology.

Todd L. Lowary should be listed as the eighth author and affiliated with the Department of Chemistry, University of Alberta, Edmonton, Alberta, Canada T6G 2G2, Institute of Biological Chemistry, Academia Sinica, Nankang, Taipei 115, Taiwan and Institute of Biochemical Sciences, National Taiwan University, Taipei 106, Taiwan. The contributions of this author are as follows: Formal Analysis, Funding Acquisition, Investigation, Project Administration, Resources, Supervision and Validation.
