# Supplementary figures and images for: Performance of novel antibodies for lipoarabinomannan to develop diagnostic tests for Mycobacterium tuberculosis
Source: PLoS One. 2022 Sep 30;17(9):e0274415. doi: 10.1371/journal.pone.0274415 (PMC9524686; doi:10.1371/journal.pone.0274415)

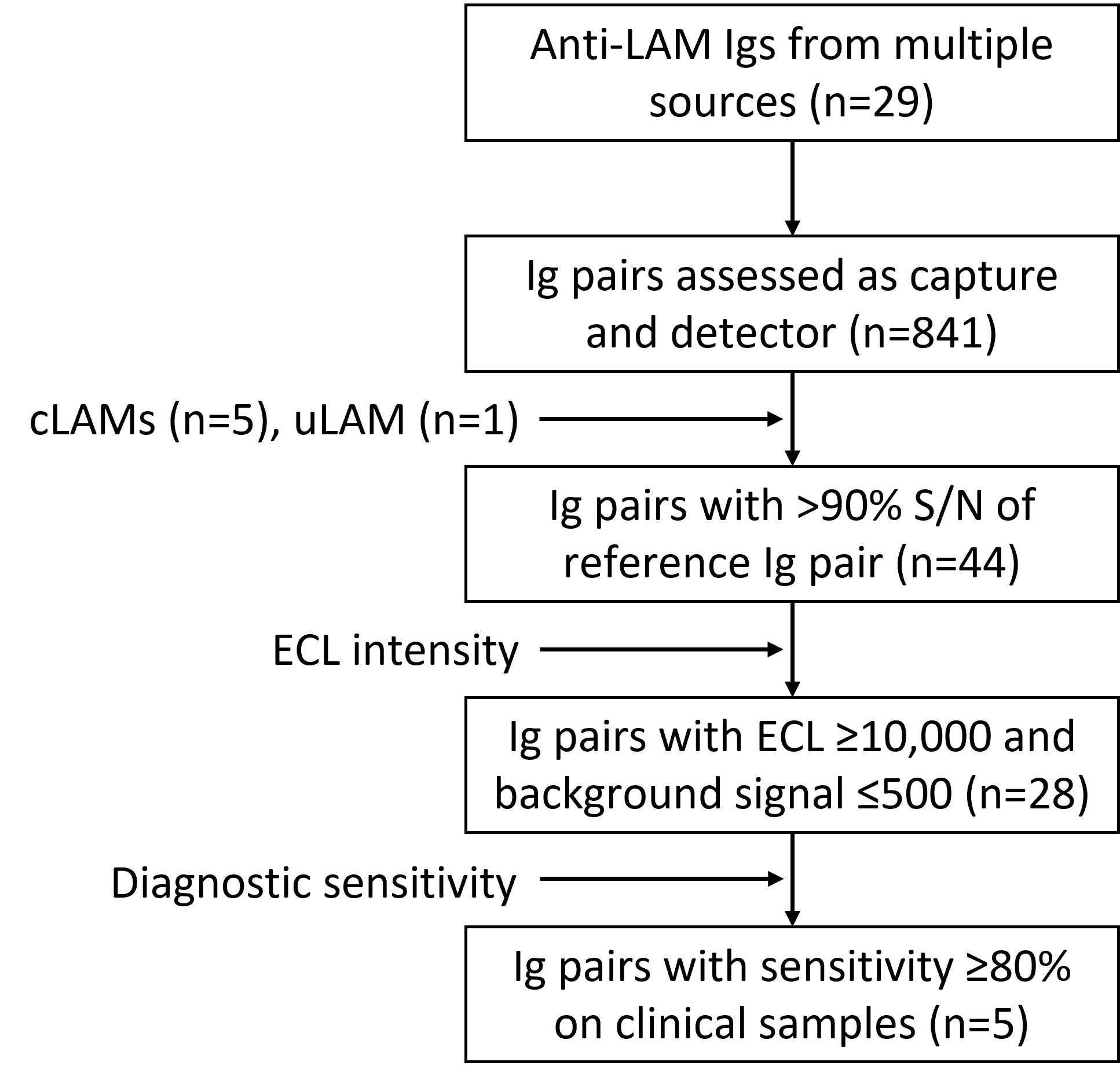

Supplement: S1 Fig — LAM, lipoarabinomannan; cLAM, cultured LAM; uLAM, urinary LAM; ECL, electrochemiluminescence; S/N, signal-over-noise. (TIF) [file pone.0274415.s001.tif]
