# Supplementary material for: Performance of novel antibodies for lipoarabinomannan to develop diagnostic tests for Mycobacterium tuberculosis
Source: PLoS One. 2022 Sep 30;17(9):e0274415. doi: 10.1371/journal.pone.0274415 (PMC9524686; doi:10.1371/journal.pone.0274415)
Supplement: S1 Table — (DOCX) [file pone.0274415.s002.docx]

S1 Table. The labeling or conjugation efficiency of the biotin and SULFO-TAG for each antibody used as the capture and detector reagents in the liquid immunoassay, respectively.

| Ig name | IgG MW (Da) | Biotin-labeled Igs | | | | | SULFO-TAG Igs | | | | |
| --- | --- | --- | --- | --- | --- | --- | --- | --- | --- | --- | --- |
|  |  | Ig (mg/mL) | Ig (mmol/mL) | Change in OD 500 nm HABA/ avidin | Biotin (mmol/mL) | mmol biotin/ mmol Ig | Ig (mg/mL) | Ig (mmol/mL) (M) | SULFO-TAG (µM) | SULFO-TAG (mmol/mL) | mmol SULFO-TAG/mmol Ig |
| A194-01 | 150000 | 1.962 | 1.31E-05 | 0.061 | 3.57E-06 | 10.9 | 1.463 | 9.76E-06 | 94.10 | 9.41E-05 | 9.6 |
| S4-20 | 150000 | 2.127 | 1.42E-05 | 0.054 | 3.19E-06 | 9.0 | 1.407 | 9.38E-06 | 50.85 | 5.09E-05 | 5.4 |
| FIND28 | 150000 | 1.202 | 8.01E-06 | 0.052 | 3.06E-06 | 15.3 | 0.929 | 6.19E-06 | 49.00 | 4.90E-05 | 7.9 |
| BTM-1 | 150000 | 1.692 | 1.13E-05 | 0.062 | 3.66E-06 | 13.0 | 1.441 | 9.61E-06 | 65.00 | 6.50E-05 | 6.8 |
| BTM-8 | 150000 | 1.836 | 1.22E-05 | 0.062 | 3.65E-06 | 11.9 | 1.602 | 1.07E-05 | 75.25 | 7.53E-05 | 7.0 |
| BJ-03 | 150000 | 0.995 | 6.63E-06 | 0.011 | 6.54E-07 | 3.9 | 1.231 | 8.21E-06 | 38.20 | 3.82E-05 | 4.7 |
| BJ-76 | 150000 | 1.359 | 9.06E-06 | 0.028 | 1.67E-06 | 7.4 | 1.170 | 7.80E-06 | 41.65 | 4.17E-05 | 5.3 |
| FDX-01 | 150000 | 1.422 | 9.48E-06 | 0.009 | 5.32E-07 | 2.2 | 1.308 | 8.72E-06 | 19.60 | 1.96E-05 | 2.2 |
| F_1D7 | 150000 | 1.726 | 1.15E-05 | 0.039 | 2.28E-06 | 7.9 | 1.482 | 9.88E-06 | 67.90 | 6.79E-05 | 6.9 |
| F_1E7 | 150000 | 2.042 | 1.36E-05 | 0.024 | 1.39E-06 | 4.1 | 1.290 | 8.60E-06 | 53.25 | 5.33E-05 | 6.2 |
| F_2B4 | 150000 | 1.368 | 9.12E-06 | 0.004 | 2.53E-07 | 1.1 | 1.440 | 9.60E-06 | 49.00 | 4.90E-05 | 5.1 |
| F_3E2 | 150000 | 1.353 | 9.02E-06 | 0.032 | 1.88E-06 | 8.3 | 1.301 | 8.67E-06 | 51.10 | 5.11E-05 | 5.9 |
| 1E7 | 150000 | 1.767 | 1.18E-05 | 0.029 | 1.70E-06 | 5.8 | 1.444 | 9.63E-06 | 14.60 | 1.46E-05 | 1.5 |
| 5E3 | 150000 | 1.130 | 7.53E-06 | 0.022 | 1.28E-06 | 6.8 | 1.005 | 6.70E-06 | 10.05 | 1.01E-05 | 1.5 |
| 7H3/7K3 | 150000 | 1.229 | 8.19E-06 | 0.038 | 2.24E-06 | 10.9 | 1.057 | 7.05E-06 | 34.00 | 3.40E-05 | 4.8 |
| 11H2/11K1 | 150000 | 0.975 | 6.50E-06 | 0.025 | 1.49E-06 | 9.2 | 0.884 | 5.89E-06 | 24.55 | 2.46E-05 | 4.2 |
| 15H3/15K3 | 150000 | 0.820 | 5.46E-06 | 0.034 | 1.99E-06 | 14.6 | 0.686 | 4.57E-06 | 19.15 | 1.92E-05 | 4.2 |
| 16H2/16K1 | 150000 | 1.005 | 6.70E-06 | 0.029 | 1.69E-06 | 10.1 | 0.855 | 5.70E-06 | 30.80 | 3.08E-05 | 5.4 |
| 17H2/17K3 | 150000 | 0.898 | 5.98E-06 | 0.020 | 1.20E-06 | 8.0 | 0.858 | 5.72E-06 | 21.10 | 2.11E-05 | 3.7 |
| 18H2/18K2 | 150000 | 1.001 | 6.67E-06 | 0.024 | 1.39E-06 | 8.3 | 0.949 | 6.33E-06 | 27.65 | 2.77E-05 | 4.4 |
| 20H3/20K2 | 150000 | 1.098 | 7.32E-06 | 0.037 | 2.15E-06 | 11.7 | 0.995 | 6.63E-06 | 28.55 | 2.86E-05 | 4.3 |
| 52H3/52K2 | 150000 | 0.629 | 4.19E-06 | 0.025 | 1.48E-06 | 14.1 | 0.567 | 3.78E-06 | 11.10 | 1.11E-05 | 2.9 |
| 79H2/79K2 | 150000 | 1.044 | 6.96E-06 | 0.032 | 1.87E-06 | 10.7 | 0.921 | 6.14E-06 | 22.15 | 2.22E-05 | 3.6 |
| 90H3/90K3 | 150000 | 0.675 | 4.50E-06 | 0.028 | 1.65E-06 | 14.7 | 0.558 | 3.72E-06 | 18.40 | 1.84E-05 | 4.9 |
| MCD024 Fab | 50000 | 0.752 | 1.50E-05 | 0.028 | 1.66E-06 | 4.4 | 0.646 | 1.29E-05 | 27.85 | 2.79E-05 | 2.2 |
| MCD024 Fab2 | 100000 | 0.787 | 7.87E-06 | 0.025 | 1.50E-06 | 7.6 | 0.770 | 7.70E-06 | 18.75 | 1.88E-05 | 2.4 |
| MCD022 Fab | 50000 | 1.061 | 2.12E-05 | 0.029 | 1.70E-06 | 3.2 | 0.759 | 1.52E-05 | 29.75 | 2.98E-05 | 2.0 |
| MCD022 Fab2 | 100000 | 1.001 | 1.00E-05 | 0.024 | 1.38E-06 | 5.5 | 0.674 | 6.74E-06 | 13.75 | 1.38E-05 | 2.0 |
|  |  |  |  |  | Average | 8.6 |  |  |  | Average | 4.5 |
