# Supplementary material for: Performance of novel antibodies for lipoarabinomannan to develop diagnostic tests for Mycobacterium tuberculosis
Source: PLoS One. 2022 Sep 30;17(9):e0274415. doi: 10.1371/journal.pone.0274415 (PMC9524686; doi:10.1371/journal.pone.0274415)
Supplement: S7 Table — (DOCX) [file pone.0274415.s008.docx]

S7 Table. Lineage 3 S / N ratio vs reference antibody pair.

|  |  | Detector antibody | | | | | | | | | | | | | | | | | | | | | | | | | | | | |
| --- | --- | --- | --- | --- | --- | --- | --- | --- | --- | --- | --- | --- | --- | --- | --- | --- | --- | --- | --- | --- | --- | --- | --- | --- | --- | --- | --- | --- | --- | --- |
|  |  | 11H2/  11K1 | 15H3/  15K3 | 16H2/  16K1 | 17H2/  17K3 | 18H2/  18K2 | 1E7 | 20H3/  20K2 | 52H3/  52K2 | 5E3 | 79H2/  79K2 | 7H3/  7K3 | 90H3/  90K3 | A194  -01 | BJ  -03 | BJ  -76 | BTM  -1 | BTM  -8 | F-1  D7 | F-1  E7 | F  -2B4 | F-3  E2 | FDX  -01 | FIND  28 | KI24 | MCD  022 Fab | MCD  022  Fab2 | MCD  024  Fab | MCD  024  Fab2 | S4-20 |
| Capture antibody | 11H2/11K1 | 3 | 1 | 1 | 1 | 1 | 14 | 1 | 4 | 6 | 2 | 1 | 4 | 11 | 2 | 5 | 7 | 3 | 1 | 1 | 1 | 1 | 5 | 5 | 12 | 10 | 10 | 14 | 8 | 41 |
|  | 15H3/15K3 | 7 | 1 | 1 | 1 | 1 | 1 | 1 | 1 | 1 | 1 | 1 | 1 | 2 | 1 | 2 | 1 | 1 | 1 | 1 | 1 | 1 | 1 | 1 | 1 | 2 | 1 | 2 | 1 | 2 |
|  | 16H2/16K1 | 13 | 2 | 1 | 1 | 1 | 7 | 1 | 4 | 13 | 2 | 1 | 5 | 27 | 3 | 9 | 5 | 3 | 1 | 1 | 1 | 1 | 5 | 3 | 8 | 18 | 17 | 23 | 8 | 51 |
|  | 17H2/17K3 | 1 | 1 | 1 | 1 | 1 | 2 | 1 | 1 | 2 | 1 | 1 | 1 | 3 | 1 | 2 | 2 | 1 | 1 | 1 | 1 | 1 | 2 | 2 | 2 | 3 | 3 | 3 | 0 | 5 |
|  | 18H2/18K2 | 2 | 1 | 1 | 1 | 1 | 10 | 1 | 4 | 8 | 2 | 1 | 4 | 22 | 2 | 6 | 6 | 3 | 1 | 1 | 1 | 1 | 5 | 4 | 12 | 4 | 9 | 10 | 1 | 33 |
|  | 1E7 | 8 | 4 | 1 | 2 | 3 | 12 | 2 | 2 | 8 | 2 | 1 | 2 | 20 | 6 | 36 | 26 | 11 | 1 | 1 | 1 | 1 | 19 | 8 | 26 | 12 | 22 | 54 | 28 | 207 |
|  | 20H3/20K2 | 1 | 1 | 1 | 1 | 1 | 3 | 1 | 2 | 3 | 1 | 1 | 2 | 7 | 2 | 2 | 2 | 2 | 1 | 1 | 1 | 1 | **-** | 2 | 4 | 2 | 5 | 4 | 0 | 9 |
|  | 52H3/52K2 | 2 | 2 | 1 | 1 | 1 | 3 | 2 | 2 | 2 | 1 | 1 | 2 | 5 | 2 | 2 | 3 | 2 | 2 | 2 | 1 | 2 | 2 | 2 | 4 | 3 | 3 | 4 | 0 | 7 |
|  | 5E3 | 7 | 4 | 2 | 3 | 4 | 7 | 3 | 2 | 4 | 2 | 2 | 2 | 10 | 14 | 65 | 41 | 19 | 1 | 1 | 1 | 1 | 49 | 13 | 35 | 7 | 8 | 69 | 52 | 279 |
|  | 79H2/79K2 | 1 | 1 | 1 | 1 | 1 | 3 | 1 | 1 | 1 | 1 | 1 | 2 | 3 | 1 | 1 | 1 | 1 | 1 | 1 | 1 | 1 | 1 | 1 | 2 | 1 | 2 | 2 | 0 | 3 |
|  | 7H3/7K3 | 16 | 1 | 1 | 1 | 1 | 2 | 1 | 2 | 3 | 1 | 1 | 2 | 28 | 1 | 2 | 2 | 1 | 1 | 1 | 1 | 1 | 2 | 2 | 3 | 5 | 4 | 5 | 2 | 7 |
|  | 90H3/90K3 | 2 | 2 | 1 | 1 | 1 | 3 | 1 | 2 | 2 | 1 | 1 | 2 | 5 | 2 | 2 | 3 | 2 | 1 | 1 | 1 | 1 | 2 | 3 | 6 | 2 | 3 | 4 | 0 | 11 |
|  | A194-01 | 3 | 2 | 1 | 2 | 2 | 4 | 2 | 2 | 3 | 1 | 1 | 1 | 4 | 4 | 12 | 23 | 9 | 2 | 1 | 1 | 1 | 7 | 8 | 16 | 3 | 6 | 35 | 17 | 91 |
|  | BJ-03 | 8 | 6 | 1 | 1 | 2 | 16 | 1 | 4 | 25 | 1 | 1 | 6 | 47 | 4 | 9 | 10 | 5 | 1 | 1 | 1 | 1 | 5 | 4 | 14 | 18 | 15 | 35 | 26 | 117 |
|  | BJ-76 | 9 | 7 | 1 | 1 | 2 | 25 | 1 | 4 | 28 | 1 | 1 | 6 | 52 | 4 | 8 | 8 | 3 | 1 | 0 | 1 | 1 | 8 | 8 | 34 | 36 | 30 | 43 | 18 | 150 |
|  | BTM-1 | 10 | 1 | 1 | 1 | 1 | 2 | 1 | 1 | 2 | 1 | 1 | 2 | 6 | 1 | 1 | 1 | 1 | 1 | 1 | 1 | 1 | 1 | 1 | 2 | 2 | 2 | 3 | 2 | 12 |
|  | BTM-8 | 2 | 1 | 1 | 1 | 1 | 1 | 1 | 1 | 1 | 1 | 1 | 1 | 4 | 1 | 2 | 1 | 1 | 1 | 1 | 1 | 1 | 2 | 1 | 1 | 1 | 2 | 2 | 1 | 3 |
|  | F-1D7 | 1 | 1 | 1 | 1 | 1 | 2 | 1 | 1 | 1 | 1 | 1 | 1 | 1 | 1 | 1 | 1 | 1 | 1 | 1 | 1 | 1 | 1 | 1 | 1 | 1 | 1 | 1 | 1 | 1 |
|  | F-1E7 | 1 | 1 | 1 | 1 | 1 | 2 | 1 | 1 | 1 | 1 | 1 | 1 | 1 | 1 | 1 | 1 | 1 | 1 | 0 | 1 | 1 | 1 | 1 | 1 | 1 | 1 | 1 | 1 | 1 |
|  | F-2B4 | 1 | 1 | 1 | 1 | 1 | 2 | 1 | 2 | 1 | 1 | 2 | 1 | 1 | 1 | 1 | 1 | 1 | 1 | 0 | 1 | 1 | 1 | 1 | 1 | 1 | 1 | 1 | 1 | 1 |
|  | F-3E2 | 1 | 1 | 1 | 1 | 1 | 1 | 1 | 1 | 1 | 1 | 1 | 1 | 1 | 1 | 1 | 1 | 1 | 1 | 1 | 1 | 1 | 1 | 1 | 1 | 1 | 1 | 1 | 1 | 1 |
|  | FDX-01 | 8 | 6 | 1 | 1 | 2 | 29 | 2 | 9 | 23 | 3 | 1 | 20 | 70 | 4 | 13 | 7 | 4 | 1 | 1 | 1 | 1 | 6 | 6 | 21 | 44 | 29 | 26 | 17 | 104 |
|  | FIND28 | 4 | 3 | 1 | 2 | 1 | 10 | 2 | 6 | 6 | 3 | 2 | 11 | 39 | 2 | 4 | 3 | 2 | 1 | 0 | 1 | 2 | 3 | 3 | 9 | 17 | 11 | 11 | 4 | 26 |
|  | MCD022 Fab | 3 | 2 | 1 | 1 | 2 | 5 | 1 | 2 | 3 | 1 | 1 | 2 | 11 | 3 | 9 | 10 | 4 | 1 | 1 | 1 | 1 | 6 | 8 | 19 | 3 | 6 | 25 | 1 | 74 |
|  | MCD022 Fab2 | 3 | 2 | 1 | 1 | 1 | 4 | 1 | 2 | 3 | 1 | 1 | 1 | 6 | 2 | 11 | 8 | 6 | 1 | 1 | 1 | 1 | 8 | 2 | 16 | 2 | 2 | 3 | 2 | 132 |
|  | MCD024 Fab | 15 | 8 | 1 | 2 | 2 | 26 | 2 | 6 | 32 | 2 | 1 | 12 | 216 | 7 | 13 | 15 | 6 | 1 | 1 | 1 | 1 | 12 | 12 | 40 | 7 | 39 | 18 | 1 | 88 |
|  | MCD024 Fab2 | 16 | 9 | 1 | 2 | 3 | 36 | 2 | 6 | 44 | 2 | 1 | 13 | 186 | 4 | 9 | 19 | 6 | 1 | 1 | 1 | 1 | 10 | 9 | 32 | 20 | 25 | 17 | 2 | 93 |
|  | S4-20 | 17 | 9 | 1 | 2 | 2 | 27 | 2 | 6 | 28 | 2 | 1 | 11 | 100 | 7 | 16 | 10 | 4 | 1 | 1 | 1 | 1 | 14 | 13 | 26 | 24 | 29 | 15 | 9 | 95 |
