# Supplementary material for: Performance of novel antibodies for lipoarabinomannan to develop diagnostic tests for Mycobacterium tuberculosis
Source: PLoS One. 2022 Sep 30;17(9):e0274415. doi: 10.1371/journal.pone.0274415 (PMC9524686; doi:10.1371/journal.pone.0274415)
Supplement: S10 Table — (DOCX) [file pone.0274415.s011.docx]

S10 Table. Lineage 5:6 S - N ratio vs reference antibody pair.

|  |  | Detector antibody | | | | | | | | | | | | | | | | | | | | | | | | | | | | |
| --- | --- | --- | --- | --- | --- | --- | --- | --- | --- | --- | --- | --- | --- | --- | --- | --- | --- | --- | --- | --- | --- | --- | --- | --- | --- | --- | --- | --- | --- | --- |
|  |  | 11H2/  11K1 | 15H3/  15K3 | 16H2/  16K1 | 17H2/  17K3 | 18H2/  18K2 | 1E7 | 20H3/  20K2 | 52H3/  52K2 | 5E3 | 79H2/  79K2 | 7H3/  7K3 | 90H3/  90K3 | A194  -01 | BJ  -03 | BJ  -76 | BTM  -1 | BTM  -8 | F-1  D7 | F-1  E7 | F  -2B4 | F-3  E2 | FDX  -01 | FIND  28 | KI24 | MCD  022 Fab | MCD  022  Fab2 | MCD  024  Fab | MCD  024  Fab2 | S4-20 |
| Capture antibody | 11H2/11K1 | 0 | 0 | 0 | 0 | 0 | 3 | 0 | 0 | 5 | 0 | 0 | 1 | 13 | 0 | 0 | 8 | 3 | 0 | 0 | 0 | 0 | 0 | 1 | 4 | 3 | 2 | 9 | 4 | 38 |
|  | 15H3/15K3 | 0 | 0 | 0 | 0 | 0 | 0 | 0 | 0 | 0 | 0 | 0 | 0 | 0 | 0 | 0 | 0 | 0 | 0 | 0 | 0 | 0 | 0 | 0 | 0 | 0 | 0 | 0 | 0 | 1 |
|  | 16H2/16K1 | 0 | 0 | 0 | 0 | 0 | 2 | 0 | 0 | 6 | 0 | 0 | 1 | 20 | 0 | 0 | 10 | 3 | 0 | 0 | 0 | 0 | 0 | 1 | 3 | 3 | 3 | 11 | 4 | 42 |
|  | 17H2/17K3 | 0 | 0 | 0 | 0 | 0 | 0 | 0 | 0 | 0 | 0 | 0 | 0 | 0 | 0 | 0 | 0 | 0 | 0 | 0 | 0 | 0 | 0 | 0 | 0 | 0 | 0 | 1 | 0 | 0 |
|  | 18H2/18K2 | 1 | 0 | 0 | 0 | 0 | 3 | 0 | 0 | 4 | 0 | 0 | 1 | 2 | 0 | 0 | 9 | 3 | 0 | 0 | 0 | 0 | 0 | 1 | 4 | 2 | 2 | 8 | 4 | 31 |
|  | 1E7 | 4 | 3 | 0 | 0 | 1 | 10 | 0 | 1 | 16 | 0 | 0 | 3 | 73 | 0 | 1 | 46 | 15 | 0 | 0 | 0 | 0 | 0 | 4 | 11 | 10 | 10 | 35 | 19 | 174 |
|  | 20H3/20K2 | 0 | 0 | 0 | 0 | 0 | 1 | 0 | 0 | 1 | 0 | 0 | 0 | 3 | 0 | 0 | 2 | 1 | 0 | 0 | 0 | 0 | **-** | 0 | 1 | 1 | 0 | 3 | 1 | 8 |
|  | 52H3/52K2 | 0 | 0 | 0 | 0 | 0 | 0 | 0 | 0 | 0 | 0 | 0 | 0 | 3 | 0 | 0 | 0 | 0 | 0 | 0 | 0 | 0 | 0 | 0 | 1 | 0 | 0 | 1 | 0 | 2 |
|  | 5E3 | 8 | 6 | 0 | 0 | 1 | 15 | 0 | 2 | 23 | 0 | 0 | 3 | 94 | 0 | 1 | 55 | 18 | 0 | 0 | 0 | 0 | 0 | 7 | 20 | 12 | 12 | 39 | 23 | 191 |
|  | 79H2/79K2 | 0 | 0 | 0 | 0 | 0 | 0 | 0 | 0 | 0 | 0 | 0 | 0 | 1 | 0 | 0 | 0 | 0 | 0 | 0 | 0 | 0 | 0 | 0 | 0 | 0 | 0 | 0 | 0 | 0 |
|  | 7H3/7K3 | 0 | 0 | 0 | 0 | 0 | 1 | 0 | 0 | 2 | 0 | 0 | 0 | 7 | 0 | 0 | 2 | 0 | 0 | 0 | 0 | 0 | 0 | 1 | 1 | 1 | 1 | 5 | 1 | 30 |
|  | 90H3/90K3 | 0 | 0 | 0 | 0 | 0 | 1 | 0 | 0 | 1 | 0 | 0 | 0 | 4 | 0 | 0 | 1 | 0 | 0 | 0 | 0 | 0 | 0 | 0 | 1 | 1 | 0 | 1 | 0 | 4 |
|  | A194-01 | 1 | 1 | 0 | 0 | 0 | 5 | 0 | 1 | 5 | 0 | 0 | 1 | 24 | 1 | 4 | 9 | 3 | 0 | 0 | 0 | 0 | 1 | 2 | 7 | 3 | 3 | 11 | 4 | 41 |
|  | BJ-03 | 2 | 2 | 0 | 0 | 0 | 5 | 0 | 0 | 8 | 0 | 0 | 1 | 30 | 1 | 13 | 18 | 7 | 0 | 0 | 0 | 0 | 2 | 2 | 7 | 3 | 2 | 14 | 8 | 59 |
|  | BJ-76 | 5 | 5 | 0 | 0 | 1 | 18 | 0 | 1 | 23 | 0 | 0 | 1 | 56 | 3 | 29 | 38 | 14 | 0 | 0 | 0 | 0 | 6 | 9 | 27 | 10 | 7 | 43 | 23 | 208 |
|  | BTM-1 | 1 | 0 | 0 | 0 | 0 | 1 | 0 | 0 | 2 | 0 | 0 | 0 | 5 | 0 | 0 | 3 | 1 | 0 | 0 | 0 | 0 | 0 | 1 | 2 | 1 | 1 | 4 | 2 | 0 |
|  | BTM-8 | 0 | 0 | 0 | 0 | 0 | 1 | 0 | 0 | 1 | 0 | 0 | 0 | 3 | 0 | 0 | 2 | 1 | 0 | 0 | 0 | 0 | 0 | 0 | 1 | 1 | 0 | 3 | 1 | 11 |
|  | F-1D7 | 0 | 0 | 0 | 0 | 0 | 0 | 0 | 0 | 0 | 0 | 0 | 0 | 0 | 0 | 0 | 0 | 0 | 0 | 0 | 0 | 0 | 0 | 0 | 0 | 0 | 0 | 0 | 0 | 0 |
|  | F-1E7 | 0 | 0 | 0 | 0 | 0 | 0 | 0 | 0 | 0 | 0 | 0 | 0 | 0 | 0 | 0 | 0 | 0 | 0 | 0 | 0 | 0 | 0 | 0 | 0 | 0 | 0 | 0 | 0 | 1 |
|  | F-2B4 | 0 | 0 | 0 | 0 | 0 | 0 | 0 | 0 | 0 | 0 | 0 | 0 | 0 | 0 | 0 | 0 | 0 | 0 | 0 | -2 | 0 | 0 | 0 | 0 | 0 | 0 | 0 | 0 | 0 |
|  | F-3E2 | 0 | 0 | 0 | 0 | 0 | 0 | 0 | 0 | 0 | 0 | 0 | 0 | 0 | 0 | 0 | 0 | 0 | 0 | 0 | 0 | 0 | 0 | 0 | 0 | 0 | 0 | 0 | 0 | 0 |
|  | FDX-01 | 2 | 2 | 0 | 0 | 0 | 7 | 0 | 1 | 10 | 0 | 0 | 2 | 51 | 1 | 16 | 16 | 6 | 0 | 0 | 0 | 0 | 2 | 4 | 16 | 7 | 5 | 22 | 10 | 94 |
|  | FIND28 | 1 | 0 | 0 | 0 | 0 | 2 | 0 | 0 | 3 | 0 | 0 | 1 | 13 | 0 | 4 | 4 | 2 | 0 | 0 | 0 | 0 | 0 | 1 | 5 | 2 | 1 | 8 | 2 | 24 |
|  | MCD022 Fab | 1 | 1 | 0 | 0 | 0 | 5 | 0 | 1 | 5 | 0 | 0 | 2 | 6 | 0 | 0 | 0 | 0 | 0 | 0 | 0 | 0 | 0 | 0 | 8 | 4 | 4 | 11 | 4 | 1 |
|  | MCD022 Fab2 | 1 | 1 | 0 | 0 | 0 | 5 | 0 | 0 | 6 | 0 | 0 | 1 | 3 | 0 | 0 | 0 | 0 | 0 | 0 | 0 | 0 | 0 | 0 | 8 | 3 | 3 | 15 | 6 | 2 |
|  | MCD024 Fab | 7 | 4 | 0 | 0 | 1 | 16 | 0 | 1 | 16 | 0 | 0 | 2 | 55 | 0 | 0 | 42 | 16 | 0 | 0 | 0 | 0 | 0 | 5 | 24 | 9 | 7 | 29 | 17 | 133 |
|  | MCD024 Fab2 | 5 | 3 | 0 | 0 | 1 | 13 | 0 | 0 | 17 | 0 | 0 | 2 | 57 | 0 | 0 | 35 | 12 | 0 | 0 | 0 | 0 | 0 | 6 | 21 | 9 | 9 | 25 | 15 | 129 |
|  | S4-20 | 8 | 5 | 0 | 0 | 1 | 17 | 0 | 1 | 20 | 0 | 0 | 2 | 100 | 6 | 44 | 49 | 18 | 0 | 0 | 0 | 0 | 10 | 14 | 22 | 10 | 9 | 25 | 16 | 195 |
