# Supplementary material for: Performance of novel antibodies for lipoarabinomannan to develop diagnostic tests for Mycobacterium tuberculosis
Source: PLoS One. 2022 Sep 30;17(9):e0274415. doi: 10.1371/journal.pone.0274415 (PMC9524686; doi:10.1371/journal.pone.0274415)
Supplement: S15 Table — (DOCX) [file pone.0274415.s016.docx]

S15 Table. uLAM S/N vs reference antibody pair.

|  |  | Detector Antibody | | | | | | | | | | | | | | | | | | | | | | | | | | | | |
| --- | --- | --- | --- | --- | --- | --- | --- | --- | --- | --- | --- | --- | --- | --- | --- | --- | --- | --- | --- | --- | --- | --- | --- | --- | --- | --- | --- | --- | --- | --- |
|  |  | 11H2/  11K1 | 15H3/  15K3 | 16H2/  16K1 | 17H2/  17K3 | 18H2/  18K2 | 1E7 | 20H3/  20K2 | 52H3/  52K2 | 5E3 | 79H2/  79K2 | 7H3/  7K3 | 90H3/  90K3 | A194  -01 | BJ  -03 | BJ  -76 | BTM  -1 | BTM  -8 | F-1  D7 | F-1  E7 | F  -2B4 | F-3  E2 | FDX  -01 | FIND  28 | KI24 | MCD  022 Fab | MCD  022  Fab2 | MCD  024  Fab | MCD  024  Fab2 | S4-20 |
| Capture antibody | 11H2/11K1 | 2 | 2 | 2 | 2 | 2 | 122 | 2 | 43 | 21 | 11 | 2 | 34 | 130 | 2 | 3 | 13 | 6 | 1 | 2 | 1 | 1 | 14 | 33 | 94 | 65 | 57 | 5 | 5 | 19 |
|  | 15H3/15K3 | 1 | 2 | 1 | 1 | 2 | 7 | 2 | 4 | 2 | 2 | 2 | 4 | 15 | 2 | 2 | 1 | 2 | 2 | 2 | 1 | 2 | 2 | 3 | 7 | 5 | 4 | 2 | 2 | 2 |
|  | 16H2/16K1 | 2 | 4 | 2 | 2 | 2 | 25 | 2 | 48 | 27 | 21 | 2 | 52 | 314 | 2 | 2 | 2 | 3 | 2 | 1 | 1 | 2 | 2 | 7 | 23 | 108 | 34 | 2 | 3 | 3 |
|  | 17H2/17K3 | 2 | 2 | 2 | 2 | 2 | 4 | 2 | 5 | 2 | 3 | 1 | 6 | 4 | 1 | 2 | 2 | 2 | 1 | 1 | 2 | 1 | 1 | 1 | 3 | 5 | 2 | 2 | 1 | 2 |
|  | 18H2/18K2 | 2 | 2 | 2 | 2 | 2 | 103 | 2 | 34 | 13 | 9 | 2 | 33 | 137 | 2 | 3 | 7 | 4 | 2 | 2 | 1 | 2 | 11 | 13 | 62 | 50 | 46 | 4 | 3 | 10 |
|  | 1E7 | 13 | 17 | 2 | 5 | 12 | 73 | 5 | 7 | 3 | 3 | 2 | 7 | 112 | 2 | 12 | 18 | 11 | 2 | 2 | 1 | 1 | 30 | 19 | 66 | 42 | 111 | 16 | 14 | 63 |
|  | 20H3/20K2 | 1 | 2 | 2 | 2 | 2 | 14 | 1 | 11 | 6 | 3 | 1 | 16 | 43 | 2 | 1 | 2 | 2 | 1 | 2 | 1 | 1 | **-** | 2 | 7 | 12 | 5 | 2 | 1 | 3 |
|  | 52H3/52K2 | 4 | 5 | 1 | 2 | 2 | 56 | 2 | 15 | 6 | 5 | 2 | 18 | 94 | 2 | 2 | 3 | 3 | 2 | 2 | 1 | 2 | 7 | 11 | 55 | 34 | 29 | 2 | 2 | 4 |
|  | 5E3 | 48 | 33 | 4 | 39 | 64 | 43 | 38 | 12 | 6 | 4 | 12 | 6 | 82 | 2 | 14 | 37 | 22 | 2 | 2 | 1 | 2 | 45 | 135 | 304 | 42 | 51 | 28 | 25 | 102 |
|  | 79H2/79K2 | 3 | 3 | 2 | 2 | 2 | 39 | 2 | 9 | 2 | 3 | 2 | 12 | 61 | 2 | 2 | 3 | 3 | 1 | 2 | 1 | 2 | 3 | 4 | 16 | 21 | 16 | 2 | 2 | 2 |
|  | 7H3/7K3 | 2 | 2 | 2 | 2 | 2 | 12 | 2 | 8 | 4 | 3 | 2 | 8 | 29 | 2 | 2 | 2 | 2 | 2 | 2 | 1 | 1 | 2 | 5 | 17 | 9 | 7 | 2 | 2 | 2 |
|  | 90H3/90K3 | 3 | 3 | 1 | 2 | 2 | 41 | 2 | 12 | 4 | 4 | 2 | 13 | 68 | 2 | 2 | 4 | 2 | 1 | 1 | 1 | 1 | 5 | 14 | 61 | 23 | 24 | 2 | 2 | 6 |
|  | A194-01 | 12 | 10 | 2 | 4 | 6 | 47 | 4 | 8 | 4 | 4 | 2 | 6 | 44 | 2 | 10 | 38 | 23 | 2 | 3 | 2 | 3 | 56 | 64 | 161 | 20 | 50 | 19 | 15 | 65 |
|  | BJ-03 | 2 | 2 | 2 | 2 | 2 | 2 | 2 | 2 | 2 | 2 | 2 | 2 | 2 | 1 | 2 | 2 | 2 | 2 | 2 | 1 | 2 | 2 | 4 | 2 | 2 | 2 | 2 | 2 | 2 |
|  | BJ-76 | 3 | 4 | 2 | 2 | 2 | 5 | 2 | 2 | 4 | 2 | 2 | 4 | 9 | 1 | 2 | 6 | 4 | 2 | 2 | 1 | 2 | 2 | 2 | 3 | 3 | 4 | 4 | 4 | 10 |
|  | BTM-1 | 3 | 3 | 2 | 2 | 2 | 3 | 2 | 2 | 3 | 2 | 2 | 3 | 16 | 2 | 2 | 2 | 2 | 2 | 1 | 1 | 2 | 2 | 3 | 3 | 4 | 5 | 3 | 3 | 3 |
|  | BTM-8 | 2 | 2 | 2 | 2 | 1 | 3 | 2 | 2 | 2 | 2 | 1 | 2 | 8 | 1 | 2 | 2 | 2 | 1 | 1 | 1 | 2 | 2 | 3 | 4 | 3 | 3 | 1 | 2 | 3 |
|  | F-1D7 | 2 | 2 | 2 | 2 | 2 | 2 | 2 | 3 | 2 | 2 | 2 | 2 | 2 | 1 | 2 | 2 | 1 | 2 | 2 | 1 | 2 | 2 | 2 | 2 | 2 | 2 | 2 | 2 | 2 |
|  | F-1E7 | 2 | 2 | 2 | 2 | 2 | 2 | 2 | 2 | 2 | 2 | 2 | 2 | 3 | 2 | 2 | 2 | 2 | 2 | 2 | 2 | 2 | 2 | 2 | 2 | 2 | 2 | 2 | 2 | 2 |
|  | F-2B4 | 2 | 2 | 2 | 2 | 2 | 2 | 2 | 2 | 2 | 2 | 2 | 2 | 26 | 2 | 2 | 2 | 2 | 2 | 2 | 2 | 2 | 2 | 2 | 2 | 2 | 2 | 2 | 2 | 2 |
|  | F-3E2 | 2 | 2 | 1 | 2 | 1 | 1 | 1 | 1 | 1 | 1 | 1 | 2 | 2 | 1 | 1 | 1 | 1 | 1 | 1 | 3 | 1 | 1 | 1 | 2 | 2 | 1 | 1 | 2 | 1 |
|  | FDX-01 | 22 | 70 | 2 | 3 | 13 | 455 | 3 | 194 | 65 | 44 | 2 | 399 | 1181 | 1 | 4 | 5 | 3 | 2 | 1 | 1 | 2 | 27 | 132 | 475 | 396 | 314 | 3 | 3 | 5 |
|  | FIND28 | 27 | 31 | 2 | 3 | 6 | 200 | 2 | 90 | 22 | 13 | 2 | 191 | 519 | 2 | 3 | 3 | 2 | 2 | 2 | 1 | 2 | 13 | 78 | 146 | 191 | 74 | 2 | 2 | 3 |
|  | MCD022 Fab | 12 | 13 | 2 | 3 | 10 | 106 | 3 | 19 | 7 | 8 | 3 | 14 | 154 | 2 | 5 | 31 | 17 | 2 | 1 | 1 | 2 | 44 | 169 | 477 | 46 | 86 | 19 | 13 | 36 |
|  | MCD022 Fab2 | 12 | 16 | 1 | 2 | 6 | 22 | 2 | 4 | 2 | 2 | 3 | 3 | 67 | 2 | 5 | 28 | 21 | 2 | 2 | 1 | 2 | 18 | 9 | 40 | 5 | 3 | 2 | 6 | 76 |
|  | MCD024 Fab | 4 | 3 | 2 | 2 | 3 | 6 | 2 | 5 | 5 | 2 | 2 | 14 | 130 | 2 | 1 | 9 | 4 | 1 | 2 | 1 | 2 | 2 | 3 | 5 | 18 | 16 | 3 | 3 | 9 |
|  | MCD024 Fab2 | 4 | 3 | 2 | 2 | 2 | 6 | 2 | 4 | 6 | 2 | 2 | 10 | 116 | 2 | 2 | 3 | 3 | 1 | 2 | 2 | 2 | 2 | 3 | 5 | 14 | 11 | 2 | 2 | 4 |
|  | S4-20 | 6 | 4 | 2 | 2 | 2 | 8 | 2 | 6 | 7 | 3 | 2 | 12 | 100 | 2 | 2 | 5 | 3 | 2 | 1 | 1 | 2 | 2 | 3 | 6 | 17 | 20 | 2 | 2 | 5 |
